# Assessment of Area-Level Disease Control and Surveillance Vulnerabilities: An Application to Visceral Leishmaniasis in Brazil

**DOI:** 10.4269/ajtmh.18-0327

**Published:** 2019-06-03

**Authors:** Victor J. del Rio Vilas, Qihua Qiu, Lucas E. Donato, Francisco Edilson F. de Lima Junior, Renato V. Alves

**Affiliations:** 1 School of Veterinary Medicine, University of Surrey, Guildford, United Kingdom;; 2 Andrew Young School of Policy Studies, Georgia State University, Atlanta, Georgia;; 3 Secretaria de Vigilância em Saúde, Ministério da Saúde (SVS-MH), Brasília, Brazil

## Abstract

The large number of activities contributing to zoonoses surveillance and control capability, on both human and animal domains, and their likely heterogeneous implementation across administrative units make assessment and comparisons of capability performance between such units a complex task. Such comparisons are important to identify gaps in capability development, which could lead to clusters of vulnerable areas, and to rank and subsequently prioritize resource allocation toward the least capable administrative units. Area-level preparedness is a multidimensional entity and, to the best of our knowledge, there is no consensus on a single comprehensive indicator, or combination of indicators, in a summary metric. We use Bayesian spatial factor analysis models to jointly estimate and rank disease control and surveillance capabilities against visceral leishmaniasis (VL) at the municipality level in Brazil. The latent level of joint capability is informed by four variables at each municipality, three reflecting efforts to monitor and control the disease in humans, and one variable informing surveillance capability on the reservoir, the domestic dog. Because of the large volume of missing data, we applied imputation techniques to allow production of comprehensive rankings. We were able to show the application of these models to this sparse dataset and present a ranked list of municipalities based on their overall VL capability. We discuss improvements to our models, and additional applications.

## INTRODUCTION

Risk classification and ranking of administrative units are frequently used to help the prioritization of resources for disease control.^1^ Although risk is a key parameter to inform resource allocation, it often fails to explicitly reflect the level of existing preparedness against the threat of concern.

Zoonoses require multidisciplinary approaches toward their control and surveillance, with multiple activities deployed at both the human and animal fronts of disease programs. The large number of activities contributing to the overall disease control capability and their likely heterogeneous implementation across the units of interest make comparisons of capability performance, for example, between administrative units, a complex task. Such comparisons are important to identify gaps in capacity development, which could lead to clusters of vulnerable areas, and to rank and subsequently prioritize resource allocation toward those areas with greatest needs. A number of studies have reported problems with the use of apparently simple approaches to area classification and rankings.^2,3^ The most salient limitations are ignoring the uncertainty and variability around the area-specific metrics; too narrow or unbalanced composite area metrics; neighboring effects, which are of greater significance if the units of interest are small areas such as municipalities; and failures to recognize and address missing values and dependencies in the data, among others.

Numerous efforts exist to measure and evaluate the health capacity of administrative areas, whether countries or subnational units. The International Health Regulations (IHR)^4^ provide an international binding framework toward the evaluation of countries’ health capacities, and have developed an exhaustive checklist of components contributing to a country’s overall health capacity. Health capacities can be either disease specific or generic, for example, legislation or surveillance, as is the case of most of the components contributing to the IHR. Whether disease specific or not, the overall health capacity of an area of interest could be synthesized into a latent indicator that pools the heterogeneous range of observable processes (e.g., surveillance timeliness) and outcome-related indicators (e.g., number of disease cases).

A number of studies have proposed methodologies to reduce and/or address the multidimensionality of health preparedness/vulnerabilities, often combining exposure (e.g., vector presence), susceptibility (e.g., population not vaccinated against a condition), and health capacity–specific indicators to inform a quantitative measure of vulnerability/preparedness to a specific threat.^5,6^ Dimensionality reduction of the multiple variables informing the components contributing to vulnerability can be achieved by weighted linear models, with weights derived from expert elicitation, regression approaches,^6^ or data reduction methods such as multi-correspondence analysis^5^ or principal component analysis. Recently, maximum entropy networks were suggested to compute composite health indicators for U.S. cities with full consideration of dependencies between the observed variables.^7^ Here, we extend on these methods by applying a spatial factor analysis (SFA) in a Bayesian framework.^3^ Our models allow addressing a number of important considerations relating to the formulation of a synthetic measure of health capacity for an unit or area of interest, namely, the need to account for uncertainty (either originating from sampling error or missing data), and for spatial correlations between neighboring areas. These adjustments correct for the instability of scores and related rankings (by means of borrowing strength among neighboring areas), account for the areas’ population sizes (as they contribute to the overall uncertainty), and allow statistical inference by formal treatment of the uncertainty. The benefits of these adjustments are best perceived in the ability of reporting statistically significant differences between the scores or ranks of the units or areas of interest.

In Brazil, visceral leishmaniasis (VL) is considered a disease of significant public health concern because of its geographical expansion, incidence with more than 3,000 new cases per year, and its severity that could lead to a mortality of > 90% in untreated patients.^8^ In Latin America, the domestic dog appears as the main reservoir of the urban cycle of the disease.^9^ As a result, VL control programs deploy surveillance and disease control activities on both the animal and human domains. In Brazil, VL control and surveillance activities are coordinated by the Ministry of Health (MOH-BRA). Based on a risk classification founded on a moving average of the number of human cases in the last 3 years, municipalities are categorized and different surveillance and control activities are indicated. A comprehensive approach to the rationalization of disease control resources would entail an assessment of risk, together with the explicit consideration of the level of coping capacities in each area or unit of interest. Studies have addressed the former for VL^10^ but, to the best of our knowledge, there have been no formal comparisons of area-level capacities in Brazil.

Here, we describe a Bayesian SFA toward the evaluation of health capacities. We do not aim to exhaustively characterize the entire VL program across Brazil, but to show the applicability of our methods to health capacity assessment. Given the zoonotic nature of VL, our capacity assessment covers both animal and human observable variables to describe an area-specific unobservable or latent capacity status, and delivers a ranking of municipalities based on their joint public and animal health capabilities against VL.

## MATERIALS AND METHODS

### Materials.

We chose the states of Ceara, in the northeast of the country, and Minas Gerais, in the southeast. Both are VL-endemic states, and combined contributed over 27% of all VL cases reported in the country in the period of study, 2007–2011. We chose this period as it encompasses the start of the collection of dog data in 2007 and the end of 2011 when the dog data template changed. We obtained data for the two aforementioned states on a number of disease control and surveillance variables as recorded by the MOH-BRA at the municipality level, our spatial unit of interest. We restricted our analyses to the two states, Ceara with 184 municipalities and Minas Gerais with 853 municipalities, to show the applicability of our methods.

For the purposes of our analyses, the latent level of joint health capacity was informed by four variables. Three variables reflected VL surveillance and control efforts in humans: 1) the average number of days from clinical onset to the date treatment was initiated (the timeliness of treatment), 2) the proportion of patients diagnosed by laboratory techniques (where the other class was clinical diagnosis), and 3) the proportion of patients who recovered from VL after receiving treatment. The fourth variable targeted the animal reservoir, specifically the areas’ dog surveillance capacity as shown by the proportion of dogs tested for VL (derived from the number of dogs tested over the canine population in each municipality). In addition, we included a fifth variable in our analysis, the municipality-specific VL risk, computed by the MOH-BRA as the moving average of human VL cases in the previous 3 years and resulting in four categories from 0 (no cases) to 4 (intense VL transmission). Risk class was inputted in our models as an ordinal categorical variable. As many municipalities did not report cases every year, we counted all municipalities with at least one case in the 5-year period to increase the number of observations in our analysis, and then, for all five variables, averaged across the period.

Human population data for each municipality for 2010 for the two states of interest were obtained from the online site of the Instituto Brasileiro de Geography and Estatistica. The canine population in each municipality was obtained from the 2015 annual rabies national vaccination campaign estimates. These population estimates are the most up-to-date and comprehensive record of the canine population in the country at the municipality level.

## METHODS

Our Bayesian SFA model assumes that the values of the observed variables *j* = 1,…,*J* for municipality *i* = 1,…,*n* can be decomposed in the following linear model:

$$Y_{\mathrm{ij}}={\text{µ}}_{j}+\lambda_{j}\cdot \delta_{i}+e_{\mathrm{ij}},$$ (1)

where µ_*j*_ is the state’s average for variable *j*, λ_*j*_ is the factor loading that quantifies the contribution of variable *j* to the different factors, δ_*i*_ is the latent vulnerability for municipality *i*, and *e*_*ij*_ is an error term. At a second level, λ_*j*_ is assigned a normal prior distribution with mean 0 and variance 1,000, except for timeliness of treatment where we used a prior with negative mean and small variance *N*(−10,1) to reflect our perception that increased time from clinical onset to treatment should be negatively correlated with capacity building, and the error term *N*(0,σ_*j*_^2^). To ensure identifiability, the model assumes that all the error terms are independently distributed, which means the manifest variables are correlated with one another only through the latent capability δ_*i*_.

To account for the possible neighboring dependence of the latent capability between municipalities, and the impact of the municipalities’ populations, under the assumption that smaller units will present greater variance in the error and factor terms, we add the spatial correlation matrix Ψ, and define *M* = diag (*m*_*i*_), where *m*_*i*_ is the square root of the population of municipality *i*. The final model is as follows:

$$\mathrm{Y|}\delta ∼Ν\left(\text{µ}+\Lambda \mathrm{\delta ,}\;{Μ}^{\mathrm{-1}}⊗\Sigma \right),$$

$$\delta ∼N\left(\text{0},\;{Μ}^{\mathrm{-1}/2}\Psi \cdot {Μ}^{\mathrm{-1}/2}\right),$$

where *Y* is an NJ × 1 vector of *Y*_*ij*_, Λ = *I*_*N*_ ⊗ λ, and Σ = diag(σ_*j*_^2^) with all the off-diagonal elements equal to 0. We assigned a conditional autoregressive prior^11^ to δ_*ij*_ so that the mean of the distribution of factors in municipality *i* is conditional to that of neighboring municipalities, and Ψ = (*I* − ω*W*)^−1^, where ω represents the degree of spatial dependence and *W* is an *N* × *N* boundary matrix with elements equal to 1 if two municipalities are adjacent to each other.

To explore the impact of arbitrary weights allocated to the manifest variables to derive a municipality-specific score, as an expert-based approach would pursue, we computed the *Z*-score for each manifest variable and compared it with the normalized squared correlation coefficients, $\rho_{j}^{2}={\text{λ}}_{j}^{2}/\left({\text{λ}}_{j}^{2}+{\text{σ}}_{j}^{2}\right)$, from our Bayesian SFA models, in effect representing the proportion of the manifest variables’ variances explained by the latent factors. At the municipality level, we summed the *Z*-scores of the manifest variables, which measure how apart a municipality vulnerability score is from the state’s average, to derive a vulnerability score for each municipality. Finally, we compared the rank of *Z*-scores with our posterior ranks.

Because the variables presented many missing values, we applied two imputation techniques. First is a “naïve” imputation where the missing value of a variable for a municipality was replaced by that of another municipality chosen randomly from the list of municipalities with complete information for that variable and year. The second imputation method incorporates the missing values into the sampler in each iteration and allows propagation of the uncertainty that stems from the missing data into that of the overall vulnerability score for each municipality (see Hogan and Tchernis^2^ for full details).

The model’s posterior distributions of the parameters of interest were estimated via Markov Chain Monte Carlo sampling, using the Gibbs sampler with one Metropolitan-Hastings step as a more efficient algorithm for the high-dimensional matrix of conditionals in our model. We ran 5,000 iterations of the sampler, and at each iteration, we rank the posterior mean of factor scores which, in effect, results in a sample, from the posterior distribution, of municipality ranks. We calculated the potential scale reduction factor for convergence.^12^ For each municipality, we computed the posterior distribution of its latent vulnerability rank and 95% credible intervals (CIs), and plotted them against the rank of the *Z*-scores. To explain the different ranking lists between the two methods, we also computed the difference between the means of the municipalities’ rank distributions to estimate the average number of ranks that two given municipalities, for each state, needed to be apart to report, with 90% confidence (i.e., the average percentage overlap among the municipalities’ rank posterior distributions), that their ranks were different. In addition, for all the municipalities, we computed the probability of being in the bottom quintile ranks, that is, those with the greatest vulnerability.

## RESULTS

**Figure 1.**
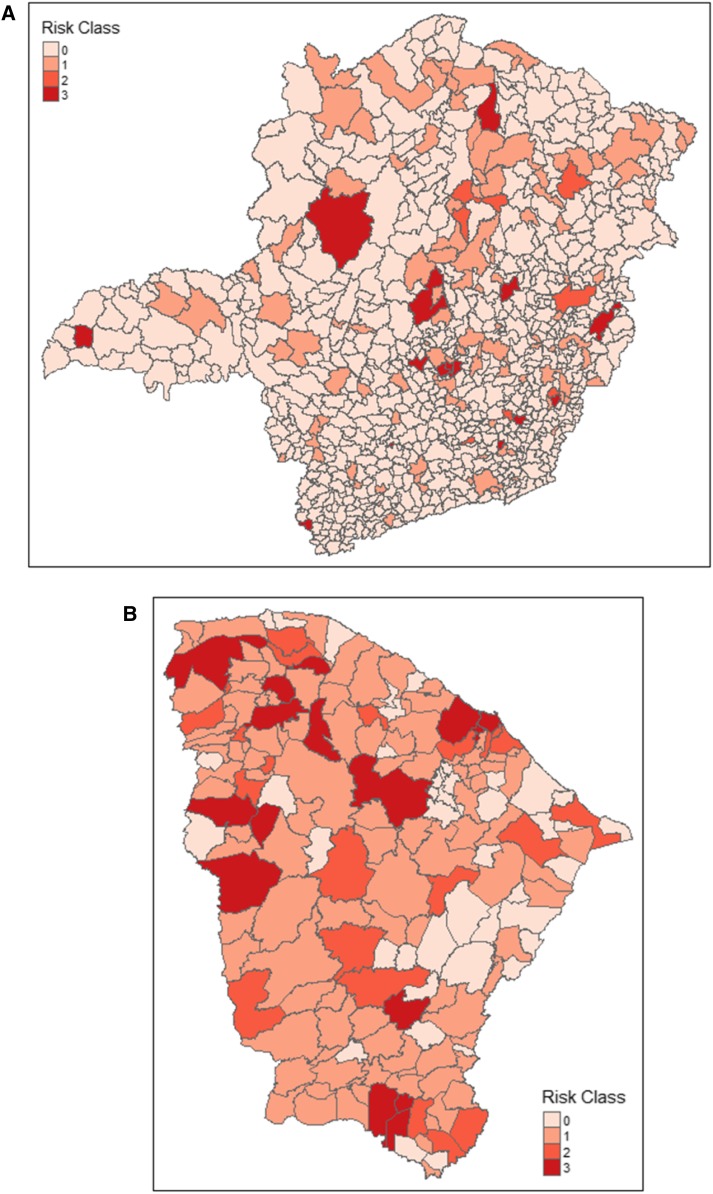
Average risk classification by municipality as applied by Brazil’s Ministry of Health. Period 2007–2011. (**A**) Minas Gerais, (**B**) Ceara. This figure appears in color at www.ajtmh.org.

In the 5-year period, Ceara reported 2,660 human VL cases. Of those, 2,037 (76.6%) recovered. The average time from clinical onset to the initiation of treatment was 42.6 days (s.d. 55.5; median 26 days). Our third manifest human vulnerability variable was the number (proportion) of patients diagnosed by laboratory techniques: 2,340 (87.9%). For Minas Gerais, there were 2,200 cases. Of these, 1,736 (78.9%) recovered. The average time from clinical onset to the initiation of treatment was 42.6 days (s.d. 53.5; median 26 days). The number (proportion) of patients diagnosed by laboratory techniques was 2,070 (94.1%) in Minas Gerais. Dog sampling for VL surveillance was inconsistent. Seven hundred fifty-four (87.3%) municipalities in Minas Gerais did not sample any dogs in the 5-year period. This compares with 19 (10.3%) municipalities in Ceara. Of those municipalities that sampled dogs at least 1 year, the average proportion of the dog population sampled was 22.1% and 12.7% for Ceara and Minas Gerais, respectively. Finally, the average risk class across the 5-year period for each municipality is shown in Figure 1 for the two states.

The potential scale reduction factor was 0.99, suggesting good convergence. Table 1 shows the results of our Bayesian SFA models, from both naïve and posterior missing imputation approaches, for the two states. In detail, we show the states’ averages (µ), and factor loadings (λ) for all observed variables. By normalizing the factor loadings for each variable, we obtain the proportion of the latent vulnerability that may be explained by each original variable. These can be interpreted as the relative contribution of the original variables to the municipalities’ ranks, and thus they can be read as prioritization criteria weights. The spatial correlation, as captured by ω, was significant for Ceara (CI not including 0), for both naïve (mean: 0.154, 95% CI: 0.150, 0.156) and posterior imputation (mean: 0.155, 95% CI: 0.153, 0.156) approaches, indicating spatial correlation. For Minas Gerais, ω was not significant, for both naïve (mean: −0.058, 95% CI: −0.25, 0.13) and posterior imputation (mean: −0.055, 95% CI: −0.25, 0.13) approaches.

**Table 1:** Results from naïve and posterior imputation approaches by state

State	Variables	Naïve imputation			Posterior imputation		
		Model parameters					
		µ	λ	Normalized squared correlation coefficient	µ	λ	Normalized squared correlation coefficient
Ceara	% cured	8.79 (8.30, 9.30)	4.04 (3.46, 4.61)	0.25 (0.24, 0.26)	8.72 (8.27, 9.22)	3.28 (2.73, 3.85)	0.27 (0.26, 0.27)
	% diagnosed	9.28 (8.75, 9.81)	4.57 (3.97, 5.23)	0.27 (0.25, 0.3)	9.21 (8.70, 9.78)	3.77 (3.22, 4.38)	0.32 (0.3, 0.33)
	% dogs sampled	5.75 (5.26, 6.29)	3.13 (2.51, 3.80)	0.14 (0.13, 0.16)	5.82 (5.34, 6.32)	2.37 (1.82, 2.95)	0.13 (0.11, 0.15)
	Timeliness	7.64 (7.07, 8.28)	3.66 (2.94, 4.43)	0.16 (0.14, 0.17)	6.81 (6.07, 7.49)	0.43 (−0.7, 1.40)	0.01 (0.0, 0.03)
	Risk class	1.60 (1.51, 1.70)	0.64 (0.53, 0.75)	0.18 (0.17, 0.18)	1.66 (1.59, 1.73)	0.50 (0.43, 0.59)	0.28 (0.27, 0.29)
Minas	% cured	1.88 (1.83, 1.94)	1.17 (1.08, 1.29)	0.29 (0.27, 0.30)	1.99 (1.93, 2.05)	0.92 (0.82, 1.03)	0.31 (0.30, 0.32)
Gerais	% diagnosed	2.13 (2.08, 2.20)	1.09 (1.00, 1.20)	0.29 (0.28, 0.31)	2.22 (2.17, 2.29)	0.88 (0.79, 0.98)	0.30 (0.29, 0.31)
	% dogs sampled	0.49 (0.45, 0.53)	0.58 (0.52, 0.64)	0.19 (0.18, 0.19)	0.77 (0.73, 0.82)	0.36 (0.28, 0.45)	0.09 (0.07, 0.12)
	Timeliness	1.65 (1.58, 1.73)	0.78 (0.65, 0.94)	0.07 (0.05, 0.09)	2.75 (2.56, 2.99)	−0.15 (−0.46, 0.13)	0 (0, 0.01)
	Risk class	0.68 (0.64, 0.73)	0.70 (0.63, 0.78)	0.17 (0.16, 0.18)	1.08 (1.05, 1.13)	0.59 (0.53, 0.66)	0.29 (0.28, 0.30)

In brackets 95% posterior interval values.

**Figure 2.**
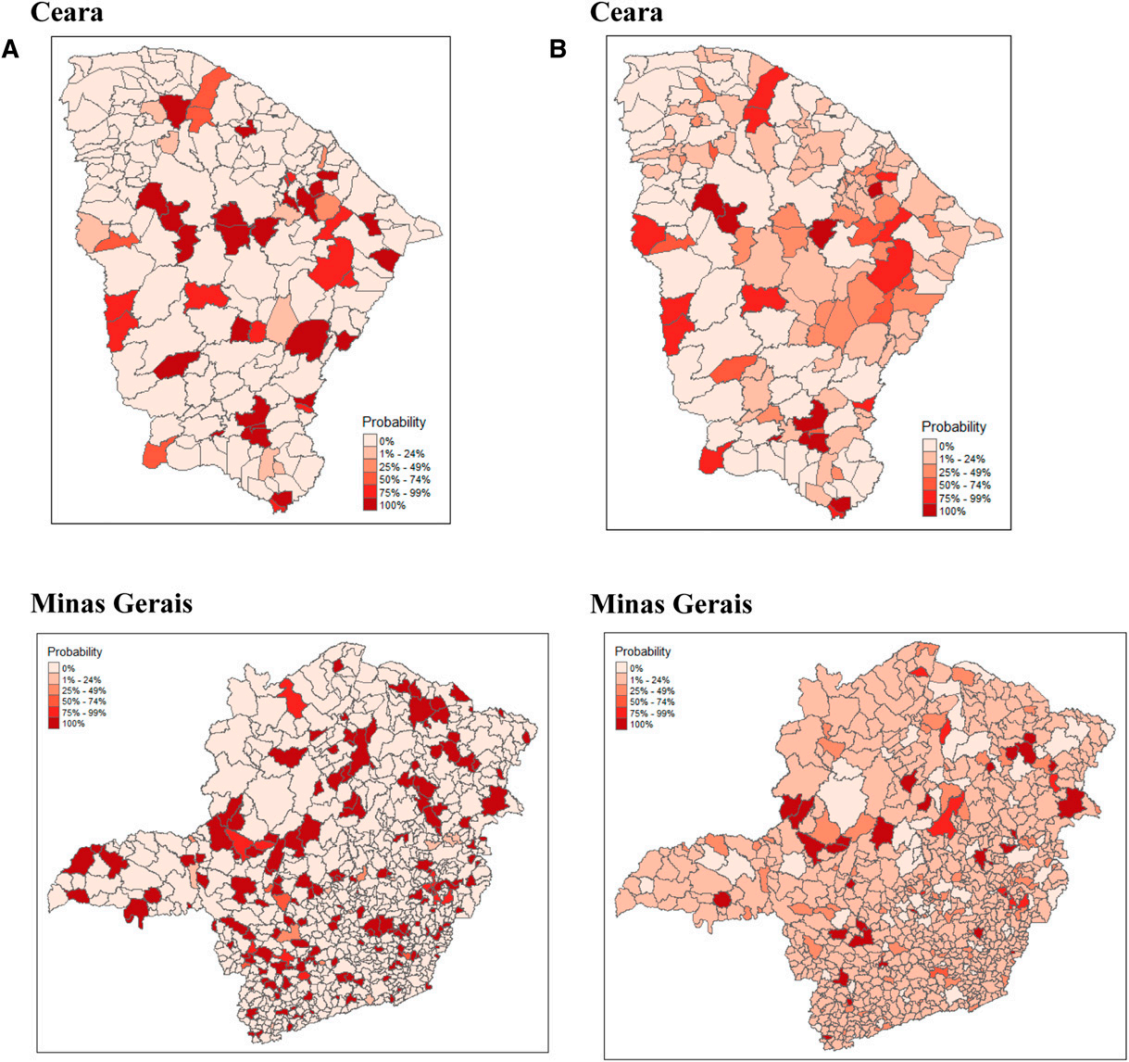
Probability of being in the least capable quintile of the posterior rank distribution for the two states. (**A**) Results from the naïve imputation method, (**B**) results from the posterior imputation method. This figure appears in color at www.ajtmh.org.

For each municipality and imputation method, we computed the posterior distribution of its vulnerability rank. Most vulnerable municipalities are those ranking at the end of the posterior rank distribution. To facilitate the identification of the municipalities with least capacity, or most vulnerable municipalities, we computed the probability of each municipality falling in the last quintile (Figure 2). Posterior imputation leads to a decrease in the number of municipalities with a probability > 0.75 of falling in the last quintile, relative to the results derived from the naïve imputation for both states. In Ceara, naïve imputation classified 34 (18.4%) municipalities with a probability > 0.75 of being in the least quintile, compared with 19 (10.3%) by posterior imputation. For Minas Gerais, naïve imputation identified 167 municipalities (19.5%) versus 35 (4.1%) by posterior imputation.

**Figure 3.**
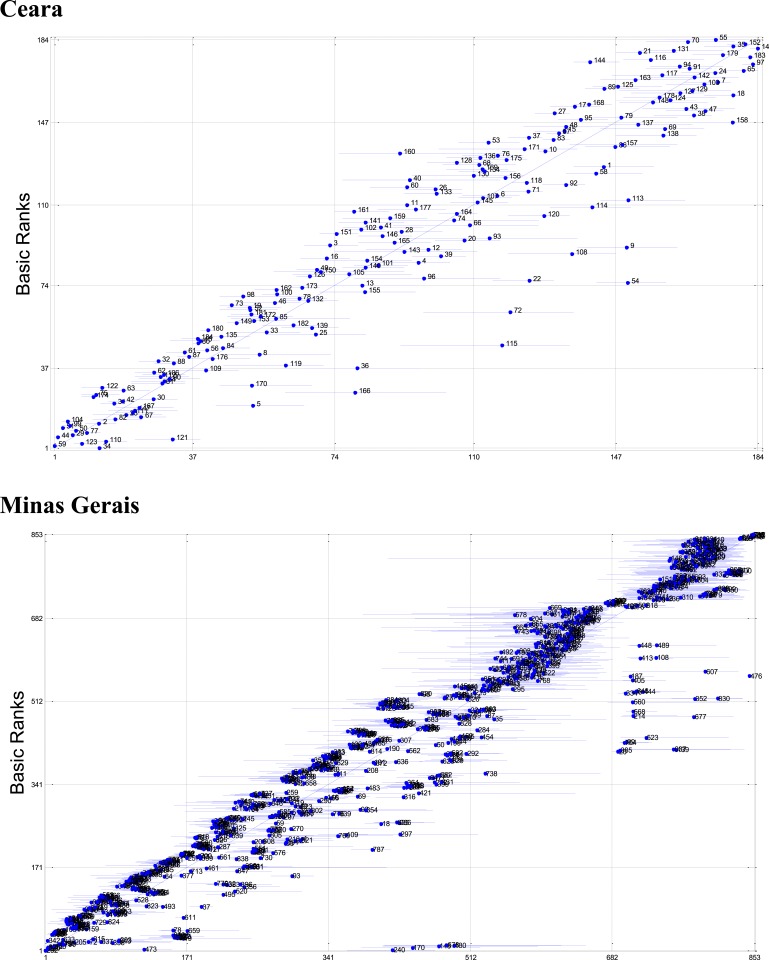
Posterior means and 99% credible intervals using naïve imputation (*x* axis), of the municipalities’ ranks, for the two states, and comparison with the municipalities’ *Z*-scores (*y* axis). This figure appears in color at www.ajtmh.org.

**Figure 4.**
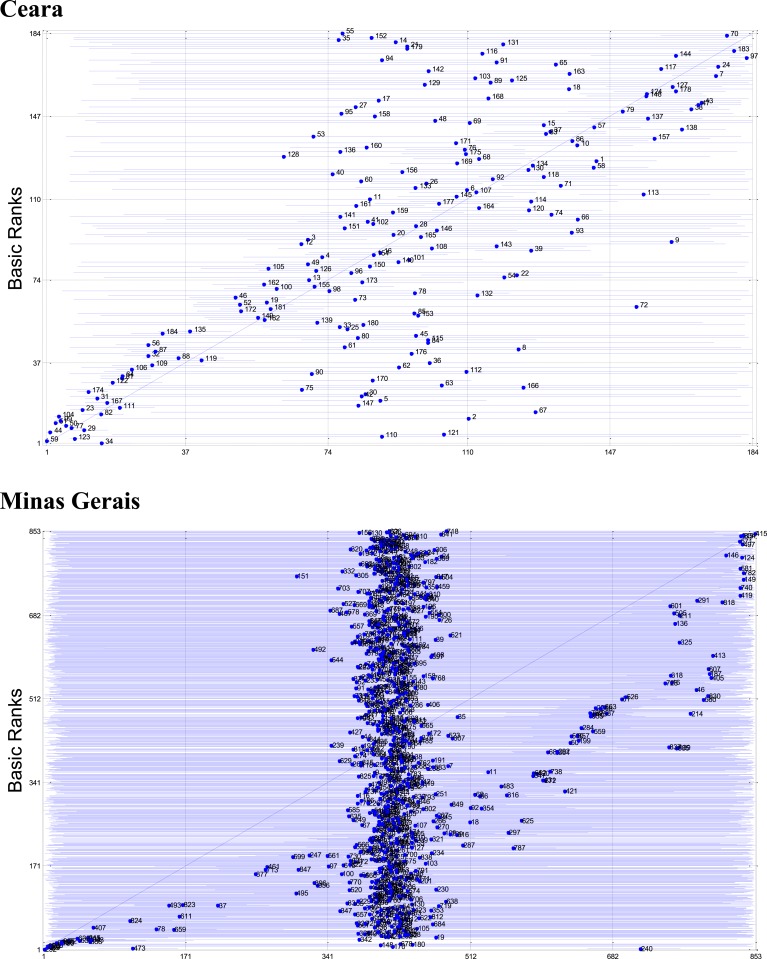
Posterior means and 99% credible intervals using posterior imputation (*x* axis), of the municipalities’ ranks, for the two states, and comparison with the municipalities’ *Z*-scores (*y* axis). This figure appears in color at www.ajtmh.org.

To understand the impact of weighting schemes on the rankings, Figure 3 shows the posterior means and 99% CIs, using naïve imputation, of the municipalities’ ranks, for the two states, and compares them with the municipalities’ *Z*-scores. We note that if the ranks derived by the two methods were the same, they would lie on the 45° line. Likewise, posterior ranks (and their CIs) entirely to the right of the 45° line indicate municipalities ranked worst by our method than by the *Z*-score approach. On average, municipalities’ ranks obtained from our models using naïve imputation were 12 and 47 ranks different from the *Z*-scores for Ceara and Minas Gerais, respectively. The same plot (Figure 4), this time with full consideration of the uncertainty derived from the missing values via posterior imputation, shows the extent of the overall uncertainty on the ranks with much larger CIs for all municipalities. This is particularly evident for Minas Gerais. On average, municipalities’ ranks obtained from our models using posterior imputation were 28 and 205 ranks different from the *Z*-scores for Ceara and Minas Gerais, respectively. The full amount of uncertainty had a clear impact on the average number of ranks required to state, with 90% confidence, that the ranks of two given municipalities were different. In the case of Ceara, the distance between the mean of two given municipalities’ vulnerability distributions was over 20 municipalities apart. In the case of Minas Gerais, this distance was over 60 municipalities apart.

## DISCUSSION

Our results show the application of Bayesian SFA models to facilitate comparisons of built-in capabilities. Our particular example shows capabilities against VL for two states of Brazil at the municipality level, where interventions are deployed. The ultimate goal of disease surveillance, and its outputs in the form of risk or capacities maps, is the provision of evidence to support targeted action and resource allocation. In our case, we were able to identify the most vulnerable municipalities where VL resources should be targeted.

The relevance of ranking reliability, or the level of instability of a ranking order due to random noise,^13^ is not commonly considered in rankings of health capacities.^14^ Failing to properly model the different sources of uncertainty, stemming from the population sizes of the municipalities, spatial covariance, and missing data (via the posterior imputation approach), leads to unstable rankings in a stochastic setting and to false certainty in a deterministic approach. As expected, posterior imputation of missing data resulted in much larger standard errors around our estimated latent vulnerabilities than those derived from naive imputation. This is due to the dynamic propagation of uncertainty by the former that samples from the full conditional posterior distribution of missing values at each iteration.

Posterior imputation of missing values, particularly for Minas Gerais where missingness was large, shows the relevance of considering the uncertainty stemming from missing data (Figure 4) and the difficulty in obtaining discriminating rankings. Given our interest in the identification of the most vulnerable municipalities, for example, those lying in the last quintile of the posterior distribution of their ranks, only 32 (3.7%) municipalities in Minas Gerais, from the model with posterior imputation, are found with their CIs entirely within this quintile. Ignoring this uncertainty, as shown by our naïve imputation results, would have mistakenly led to the conclusion that 163 (19.1%) municipalities, those with their entire CIs to the right of the 80th quintile, belonged to that “most vulnerable” class. Six times more municipalities could have been classified as vulnerable. For Ceara, where missingness was not so large, posterior imputation allowed the identification of 16 (8.7%) municipalities with their entire CIs in the last quintile of the rankings’ posterior distribution. The differences in the rankings between the two imputation methods clearly illustrate the unstability of the ranks depending on the scoring approach.

Our selection of manifest variables was informed by the opinion of VL experts, out of the reduced number of variables recorded and sufficiently populated at the municipality level. The limited number of variables at our disposal meant that we mixed outcome-related variables (% of VL cases cured) with process-related variables (e.g., timeliness of treatment) relating to some disease control capacities. Ideally, we would like to concentrate on processes and capacities upon which we can invest resources if found lacking, rather than on outcomes.

The normalized squared correlation coefficients for each manifest variable can be interpreted as their relative contribution to the latent capability.^3^ Table 1 presents the results at the state level. Timeliness from clinical inception to treatment onset contributed the least in both states, with no contribution in the case of Minas Gerais and negligible contribution for Ceara (Table 1, posterior imputation). The % of dogs sampled by the VL surveillance efforts contributed 9% and 13% of the overall capacity across Minas Gerais and Ceara, respectively.

Visceral leishmaniasis is a complex disease, and its control, a multipronged effort. We do not claim that our set of variables constitutes an exhaustive representation of these efforts, or that we have considered the most relevant variables that contribute to the municipalities’ vulnerability. This was never the intention. Other variables addressing human capacities might have been considered. The original dataset provided by the MOH-BRA contained a number of other variables, but they all described patient characteristics, for example, age and gender, and hence they would not translate immediately into program capacities. Other program activities, for example, those targeting vector control, could have been included. However, the data available were not informative and we did not consider it any further. On the animal reservoir, a number of interventions informing program capacities, specifically repellent collars and euthanasia, could have been added to our surveillance variable. However, again, the data were not available in a suitable format. Finally, we note that our results apply at the municipality level and, hence, do not capture the likely intra-municipality heterogeneity in the distribution of capacities. This level of granularity would be required for the targeted distribution of resources within municipalities. In other words, state planners are the target of our results.

### Perspectives.

Our approach is flexible to illustrate the relevance of different weighting schemes on rank orders, and the limitations of simplistic approaches to ranking. Our methods complement other current work to build evaluation frameworks for rabies capabilities,^15^ and for surveillance evaluation in general.^16^ Such models, built on simple multi-criteria decision methodologies to facilitate their development and deployment, combine multiple surveillance attributes (e.g., sensitivity) and rabies capabilities (e.g., canine vaccination) to derive area-specific scores. The accumulation of areas’ scores from the application of these evaluation frameworks would allow comparisons with Bayesian SFA models on the same data.

Many extensions to our work are possible. Visceral leishmaniasis data as provided by the MOH-BRA were patient specific and, hence, individual-related variables, for example, age, race, and type of residence (urban or rural), were available. With the specific denominator data at the municipality level, stratified analyses would have been possible. However, further fragmentation of our dataset would have resulted in larger uncertainty. In addition, temporal models with the inclusion of time-specific terms in the linear predictor component of our models, to allow the analysis of panel data, would be of interest to assess trends of capacities.

Future work should focus on extending the framework to the whole country and improving the manifest variable selection with consideration of other variables of potential interest, those informing capability deployment (e.g., installed laboratory capacity and number of health staff qualified to diagnose VL). We note that our models are able to incorporate more variables, provided they are as complete as possible and do not add more missing values to our already sparse dataset.
